# Accuracy of the Alvarado Score in Diagnosing Appendicitis Among Children Versus Adults

**DOI:** 10.7759/cureus.107159

**Published:** 2026-04-16

**Authors:** Saleh Mashhour Al Habahbeh, Abdullah A Abukaff, Ahmad Ibrahim Al-Arman, Rawan Ayyad, Abdallah Hisham Khamash, Tamara Ahmad Obeidat, Haneen Noures, Dana Fuad Eisouh, Saad Khaleel Yamin, Zaid Fawzi Elyan

**Affiliations:** 1 General and Breast Surgery, Royal Medical Services Hospitals, Amman, JOR; 2 Surgical Oncology, Royal Medical Services Hospitals, Amman, JOR; 3 General Surgery, Royal Medical Services Hospitals, Amman, JOR; 4 Radiotherapy, Military Cancer Center/Jordanian Royal Medical Services, Amman, JOR; 5 Diagnostic Radiology, Royal Medical Services Hospitals, Amman, JOR; 6 Pathology, Jordanian Royal Medical Services, Amman, JOR; 7 Colorectal Surgery, Royal Medical Services Hospitals, Amman, JOR; 8 Internal Medicine, Royal Medical Services Hospitals, Amman, JOR

**Keywords:** alvarado score, appendicitis, diagnostic accuracy, pediatric, roc curve

## Abstract

Background: The Alvarado score is widely used to risk-stratify suspected acute appendicitis; however, its diagnostic performance may vary across age groups.

Aim: To compare the diagnostic discrimination of the Alvarado score between adults (≥18 years) and children (<18 years) using receiver operating characteristic (ROC) analysis, with the primary outcome being the difference in area under the curve (AUC) between groups. Secondary analyses evaluated diagnostic performance at predefined clinical thresholds (>7 and ≥5).

Methods: This retrospective cohort study included 743 consecutive patients evaluated for suspected appendicitis. The index test was the Alvarado score derived from documented presentation findings. The reference standard was histopathological confirmation of appendicitis. Patients were categorized into children and adults. Discrimination was assessed using ROC analysis, and diagnostic performance was evaluated at predefined thresholds (high >7; equivocal ≥5), reporting sensitivity, specificity, positive predictive value (PPV), and negative predictive value (NPV). ROC curves were compared between adults and children using the DeLong test.

Results: The Alvarado score demonstrated moderate diagnostic discrimination in both groups. The area under the ROC curve (AUC) was 0.685 in adults and 0.732 in children, with no statistically significant difference between groups (DeLong p = 0.305). At the high threshold (>7), overall sensitivity was 50.5%, with 48.5% in adults and 55.7% in children, while specificity was 80.4% in adults and 78.7% in children (80.0% overall). At the equivocal threshold (≥5), sensitivity increased to 84.3% in adults, 91.4% in children, and 86.3% overall, with specificity decreasing to 25.7% in adults, 24.6% in children, and 25.4% overall. Positive predictive values ranged from 69.7% to 85.7%, while negative predictive values ranged from 43.5% to 55.6%.

Conclusions: The Alvarado score demonstrated moderate diagnostic discrimination in both adults and children, with no statistically significant difference in ROC performance between groups. At the traditional high threshold (>7), sensitivity was limited, whereas lower thresholds (≥5) increased sensitivity with reduced specificity. These findings support the use of the Alvarado score as a clinical risk stratification tool in both age groups.

## Introduction

Appendicitis is the predominant abdominal surgical emergency globally and could lead to serious medical consequences [1]. Despite its prevalence, diagnosis remains a clinical challenge, particularly because symptoms and disease progression can differ markedly across age groups. Children often present with atypical or nonspecific complaints, while adults are more likely to demonstrate the classical clinical picture, a distinction with significant implications for timely diagnosis and treatment [2-4]. Misdiagnosis or delayed diagnosis can increase the rates of perforation and negative appendectomy, underscoring the need for accurate, reproducible diagnostic tools [5].

The Alvarado score, introduced in 1986, is one of the most widely adopted clinical scoring systems for suspected appendicitis [1]. It is based on eight simple clinical and laboratory parameters, making it easy to apply in both resource-limited and high-resource settings. Over the past three decades, the score has been extensively validated and compared with other clinical prediction rules such as the Appendicitis Inflammatory Response (AIR) score and the Adult Appendicitis Score [6-8]. However, systematic reviews and prospective validation studies suggest that its diagnostic accuracy is variable, with differences noted in sensitivity, specificity, and overall discrimination depending on patient demographics and clinical setting [9,10]. Of particular concern is its performance in pediatric populations.

While the Alvarado score has been shown to achieve reasonable accuracy in adults, several studies highlight its limitations in children, where alternative scoring systems, such as the Pediatric Appendicitis Score (PAS), may provide better diagnostic utility [11-13]. For instance, evidence indicates that children may be misclassified using conventional cutoffs, resulting in missed or delayed diagnoses [14-16]. Conversely, in adults, false positives may increase unnecessary admissions or imaging studies [17-19]. Recent meta-analyses and comparative studies reinforce the need to re-examine whether a single threshold can be reliably applied across all age groups, or if age-specific cutoffs and adjunctive strategies are more appropriate [20-22].

Given these uncertainties, further evaluation is needed to clarify how the Alvarado score performs in children versus adults and whether its predictive value should guide age-tailored diagnostic strategies. This study aims to directly compare the discriminative ability and diagnostic accuracy of the Alvarado score between pediatric and adult patients with suspected appendicitis. By addressing this gap, we seek to provide evidence that may reduce unnecessary interventions in adults while minimizing delays in pediatric care.

## Materials and methods

Study design and setting

This retrospective cohort study was conducted at Queen Alia Military Hospital, Amman, Jordan, and included all consecutive patients presenting with suspected acute appendicitis between January 2022 and May 2025. Patients were stratified into two age groups: children (<18 years) and adults (≥18 years).

Ethical considerations

The requirement for obtaining informed consent was waived by the Institutional Review Board of the Royal Medical Services due to the retrospective nature of the study and the use of anonymized patient data.

Data collection

Clinical and demographic data were extracted from electronic medical records and operative reports. Variables collected included age, sex, and the individual components of the Alvarado score, including migration of pain, anorexia, nausea or vomiting, right iliac fossa tenderness, rebound tenderness, elevated temperature, leukocytosis, and left shift of neutrophils. The total Alvarado score was calculated for each patient at presentation. Radiological findings, including computed tomography (CT) and ultrasound, when available, were reviewed and categorized as inflamed appendicitis, perforated appendicitis, or non-inflamed findings. Intraoperative findings were extracted from operative reports and classified as inflamed appendix, non-inflamed appendix, perforated appendix, or other intraoperative pathology. Histopathological examination served as the reference standard for diagnosis. Patients with missing histopathological results or incomplete Alvarado score components were excluded from the final analysis.

Application of the Alvarado score

The Alvarado score was retrospectively derived for each patient using the standard 10-point scoring system based on clinical documentation at initial presentation: migration of pain (1 point), anorexia (1 point), nausea or vomiting (1 point), right lower quadrant tenderness (2 points), rebound tenderness (1 point), elevated temperature (1 point), leukocytosis (2 points), and left shift of neutrophils (1 point). Two independent clinicians calculated the Alvarado score for each patient from the medical record. Because scoring was performed retrospectively using existing clinical documentation, complete blinding to downstream diagnostic information and final diagnosis was not feasible. To evaluate the reproducibility of the retrospectively derived score, inter-rater reliability between the two clinicians was assessed using the intraclass correlation coefficient (ICC) with a two-way random-effects model for absolute agreement. Patients were categorized according to the traditional Alvarado score interpretation: low probability (1-4), equivocal probability (5-6), and high probability (7-10).

Statistical analysis

Statistical analyses were performed using R statistical software (version 4.3.0; R Foundation for Statistical Computing, Vienna, Austria). Continuous variables, including age and total Alvarado score, were reported as mean ± standard deviation (SD) and compared between groups using the Wilcoxon rank-sum test. Categorical variables were presented as frequencies and percentages, and comparisons between adults and children were performed using Pearson’s Chi-square test or Fisher’s exact test, as appropriate. The primary outcome of the study was the difference in diagnostic discrimination of the Alvarado score between adults and children, assessed using receiver operating characteristic (ROC) analysis and the area under the curve (AUC). ROC curves were generated for each age group and compared using the DeLong test. Diagnostic performance of the Alvarado score was further evaluated at predefined clinical thresholds (>7 and ≥5) by calculating sensitivity, specificity, positive predictive value (PPV), negative predictive value (NPV), and overall accuracy. Finally, multivariable logistic regression analysis was performed to evaluate the association between the total Alvarado score and age group with histopathologically confirmed appendicitis. Results were reported as odds ratios (OR) with 95% confidence intervals (CI). A two-tailed p-value <0.05 was considered statistically significant.

## Results

Of the 774 patients initially identified, 31 were excluded due to missing Alvarado score components (n = 27) or missing histopathology (n = 4), resulting in a final analytic cohort of 743 patients. The mean age of the cohort was 28.94 ± 14.45 years. Adults constituted 542/743 (72.9%) of the cohort, while 201/743 (27.1%) were children (Table 1).

**Table 1:** Descriptive analysis of demographical and clinical data of the patients Mean (SD); n (%)

Characteristic	N = 774
Age	28.94 (14.45)
Age classification	
Adult	570 (73.64%)
Childreen	204 (26.36%)
Alvarado items	
RT Iliac Fossa Pain	658 (87.04%)
Anorexia	336 (44.44%)
Nausea & Vomiting	529 (69.97%)
RT Iliac Fossa Tenderness	642 (84.92%)
Rebound Tenderness	515 (68.12%)
Elevated Temperature	131 (17.33%)
Leukocytosis	490 (64.64%)
Shift to the left	510 (67.28%)
Total Alvarado score	6.54 (2.27)
Intraoperative Finding	
Inflamed	514 (66.67%)
Non-Inflamed	216 (28.02%)
Other	8 (1.04%)
perforated	33 (4.28%)
Histopathology	
Acute appendicitis	432 (56.10%)
Acute perforative appendicitis	57 (7.40%)
Early inflamed appendix	17 (2.21%)
Non-Inflamed	253 (32.86%)
Other	11 (1.43%)
CT Findings	
Inflamed ( acute appendicitis )	376 (61.94%)
Inflamed ( perforated )	12 (1.98%)
Non-Inflamed	219 (36.08%)

Among the individual components of the Alvarado score, right iliac fossa pain was documented in 658 patients, anorexia in 336 patients, and nausea or vomiting in 529 patients. Right iliac fossa tenderness was present in 642 patients, rebound tenderness in 515 patients, elevated temperature in 131 patients, leukocytosis in 490 patients, and left shift of neutrophils in 510 patients. The mean total Alvarado score was 6.54 ± 2.27 (Table 1). Intraoperative findings demonstrated an inflamed appendix in 514 patients, a non-inflamed appendix in 216 patients, perforation in 33 patients, and other intraoperative pathology in eight patients. Histopathological examination identified acute appendicitis in 432 patients, acute perforative appendicitis in 57 patients, and early inflamed appendix in 17 patients, while 253 specimens were non-inflamed and 11 demonstrated other diagnoses (Table 1). Computed tomography was performed in 607 patients, demonstrating inflamed appendicitis in 376 cases, perforated appendicitis in 12 cases, and non-inflamed findings in 219 cases (Table 1).

Comparisons between adults and children are presented in Table 2. Anorexia was observed in 228/542 (42.1%) adults and 108/201 (53.7%) children. Nausea or vomiting occurred in 369/542 (68.1%) adults and 160/201 (79.6%) children. Elevated temperature was documented in 80/542 (14.8%) adults and 51/201 (25.4%) children. Right iliac fossa pain was present in 473/542 (87.3%) adults and 185/201 (92.0%) children, while right iliac fossa tenderness occurred in 467/542 (86.2%) adults and 175/201 (87.1%) children. Rebound tenderness was recorded in 387/542 (71.4%) adults and 128/201 (63.7%) children. Leukocytosis was present in 354/542 (65.3%) adults and 135/201 (67.2%) children, and left shift of neutrophils occurred in 363/542 (67.0%) adults and 146/201 (72.6%) children (Table 2). 

**Table 2:** Comparisons of clinical findings between adults (>18 years) and children (<18 years) in cohort ^1^Mean (SD); n (%). ^2^Wilcoxon rank sum test; Pearson’s Chi-squared test; Fisher’s exact test.

Characteristic	Adult N = 570^1^	Children N = 204^1^	p-value^2^
Alvarado items			
RT Iliac Fossa Pain	473 (85.69%)	185 (90.69%)	0.069
Anorexia	228 (41.30%)	108 (52.94%)	0.004
Nausea & Vomiting	369 (66.85%)	160 (78.43%)	0.002
RT Iliac Fossa Tenderness	467 (84.60%)	175 (85.78%)	0.7
Rebound Tenderness	387 (70.11%)	128 (62.75%)	0.054
Elevated Temperature	80 (14.49%)	51 (25.00%)	<0.001
Leukocytosis	355 (64.08%)	135 (66.18%)	0.6
Shift to the left	364 (65.70%)	146 (71.57%)	0.13
Total Alvarado score	6.43 (2.34)	6.84 (2.05)	0.064
Intraoperative Finding			0.14
Inflamed	384 (67.72%)	130 (63.73%)	
Non-Inflamed	154 (27.16%)	62 (30.39%)	
Other	8 (1.41%)	0 (0.00%)	
Perforated	21 (3.70%)	12 (5.88%)	
Histopathology			0.7
Acute appendicitis	312 (55.03%)	120 (59.11%)	
Acute perforative appendicitis	43 (7.58%)	14 (6.90%)	
Early inflamed appendix	11 (1.94%)	6 (2.96%)	
Non-Inflamed	192 (33.86%)	61 (30.05%)	
Other	9 (1.59%)	2 (0.99%)	
CT Findings			0.033
Inflamed (acute appendicitis	302 (64.53%)	74 (53.24%)	
Inflamed (perforated)	10 (2.14%)	2 (1.44%)	
Non-Inflamed	156 (33.33%)	63 (45.32%)	

The mean total Alvarado score was 6.43 ± 2.34 in adults and 6.84 ± 2.05 in children (Table 2). Intraoperative findings demonstrated inflamed appendicitis in 380/542 (70.1%) adults and 130/201 (64.7%) children, non-inflamed appendix in 146/542 (26.9%) adults and 62/201 (30.8%) children, perforated appendix in 20/542 (3.7%) adults and 12/201 (6.0%) children, and other findings in five adults (Table 2). Histopathological examination revealed acute appendicitis in 310/542 (57.2%) adults and 120/201 (59.7%) children, acute perforative appendicitis in 43/542 (7.9%) adults and 14/201 (7.0%) children, early inflamed appendix in 10/542 (1.8%) adults and 6/201 (3.0%) children, non-inflamed appendix in 179/542 (33.0%) adults and 61/201 (30.3%) children, and other diagnoses in 8 adults and 2 children (Table 2). Computed tomography demonstrated inflamed appendicitis in 301 adults and 74 children, perforated appendicitis in 10 adults and 2 children, and non-inflamed findings in 154 adults and 63 children (Table 2).

Diagnostic performance of the Alvarado score at predefined thresholds is presented in Table 3. At the high threshold (>7), the overall sensitivity was 50.5% (95% CI 46.0-55.0) and specificity 80.0% (95% CI 74.4-84.9). In adults, sensitivity was 48.5% (95% CI 43.2-53.8) and specificity 80.4% (95% CI 73.9-86.0), while in children, sensitivity was 55.7% (95% CI 47.1-64.1) and specificity 78.7% (95% CI 66.3-88.1) (Table 3). Positive predictive values were 83.4% in adults and 85.7% in children, while negative predictive values were 43.5% in adults and 43.6% in children.

**Table 3:** Diagnostic performance of the Alvarado score at high (>7) and equivocal (5) thresholds in adults and children TP: True Positive, FP: False Positive, TN: True Negative, FN: False Negative, PPV: Positive Predictive Value, NPV: Negative Predictive Value.

Group	Threshold	N	TP	FP	TN	FN	Sensitivity	Specificity	PPV	NPV	Accuracy
Adults	High (>7)	568	252	56	131	129	66.1% (61.1-70.9)	70.1% (62.9-76.5)	81.8% (77.0-86.0)	50.4% (44.1-56.6)	67.4% (63.4-71.3)
Children	High (>7)	175	91	18	35	31	74.6% (61.1-70.9)	66.0% (62.9-76.5)	83.5% (77.0-86.0)	53.0% (44.1-56.6)	72.0% (63.4-71.3)
Adults	Equivocal (5)	568	321	139	48	60	84.3% (61.1-70.9)	25.7% (62.9-76.5)	69.8% (77.0-86.0)	44.4% (44.1-56.6)	65.0% (63.4-71.3)
Children	Equivocal (5)	175	113	40	13	9	92.6% (61.1-70.9)	24.5% (62.9-76.5)	73.9% (77.0-86.0)	59.1% (44.1-56.6)	72.0% (63.4-71.3)
Overall	High (>7)	743	343	74	166	160	68.2% (61.1-70.9)	69.2% (62.9-76.5)	82.3% (77.0-86.0)	50.9% (44.1-56.6)	68.5% (63.4-71.3)
Overall	Equivocal (5)	743	434	179	61	69	86.3% (61.1-70.9)	25.4% (62.9-76.5)	70.8% (77.0-86.0)	46.9% (44.1-56.6)	66.6% (63.4-71.3)

At the equivocal threshold (≥5), the overall sensitivity increased to 86.3% (95% CI 83.0-89.2), with specificity 25.4% (95% CI 20.0-31.4). In adults, sensitivity was 84.3% (95% CI 80.1-87.9) and specificity 25.7% (95% CI 19.5-32.8), while in children, sensitivity was 91.4% (95% CI 85.5-95.5) and specificity 24.6% (95% CI 14.5-37.3) (Table 3). Positive predictive values were 69.7% in adults and 73.6% in children, and negative predictive values were 44.7% in adults and 55.6% in children (Table 3).

**Figure 1:**
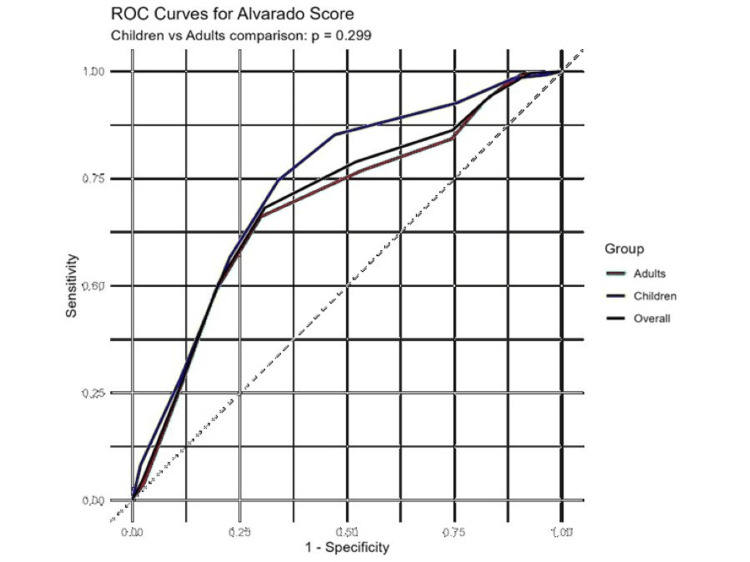
ROC curves for the Alvarado score in adults, children, and the overall cohort ROC: Receiver Operating Characteristic.

Receiver operating characteristic analysis demonstrated moderate discriminative ability of the Alvarado score (Figure 1). The area under the curve (AUC) was 0.685 for adults, 0.732 for children, and 0.698 for the overall cohort (Figure 1). Comparison of ROC curves using the DeLong test showed no statistically significant difference between adults and children (p = 0.305).

## Discussion

In our retrospective cohort of over 743 patients, we have found that the Alvarado score as a diagnostic tool showed a comparable performance between children and adults. Receiver operating characteristic analysis further demonstrated acceptable discriminative performance of the Alvarado score overall and within age subgroups. Specifically, children exhibited higher frequencies of anorexia, nausea, vomiting, and fever, contributing to higher total Alvarado scores. However, the specificity of the score was lower in children, leading to a higher risk of false-positive diagnoses if the traditional cut-off of ≥7 was applied. A key finding of this study was the low sensitivity of Alvarado score at a threshold of >7. Therefore, a score of ≥7 must be followed by imaging and clinical evaluation to avoid missing patients with appendicitis.

These findings are consistent with the original work by Alvarado (1986) [1], which first highlighted the score as a rapid bedside tool, and with later systematic reviews confirming its wide adoption but variable performance across populations [2,3]. In our study, the sensitivity of the ≥7 cut-off was moderate in both groups (60% overall), whereas the sensitivity of the equivocal threshold (≥5) exceeded 85%, at the expense of very low specificity, with negative predictive values remaining modest due to the high prevalence of appendicitis in the cohort. This pattern mirrors the results reported by Meltzer et al. (2013) [4], who showed that the score tends to perform better as a “rule-out” rather than a “rule-in” test in adults. Similarly, the WSES Jerusalem guidelines emphasize that while clinical scores such as Alvarado provide valuable triage, they should not replace imaging or surgical assessment [5]. A clinically important finding is the low sensitivity of the Alvarado score at the traditional high threshold (>7). In our cohort, sensitivity at >7 was approximately 50% overall (48.5% in adults; 55.7% in children), indicating that nearly half of histopathologically confirmed appendicitis cases would not be captured by this threshold alone. This limits the score’s role as a stand-alone ‘rule-in’ trigger for surgical intervention and underscores the need for imaging and clinical reassessment when suspicion persists despite a score ≤7. In contrast, the lower threshold (≥5) improved sensitivity substantially in both adults and children but at the cost of very low specificity, reinforcing that the score is best interpreted as a triage/risk-stratification tool rather than a definitive diagnostic test.

A key finding in our cohort was the high negative appendectomy rate (~33%), consistent with the limited specificity observed across Alvarado score thresholds. This observation aligns with prior evaluations of risk prediction models, which consistently demonstrate that imaging or combined scores improve diagnostic accuracy compared with Alvarado alone [6,7]. For example, Kong et al. (2014) [18] found that the Adult Appendicitis Score [8,14] and Appendicitis Inflammatory Response (AIR) score [22] have both shown superior specificity in adults, suggesting that Alvarado may no longer be the optimal stand-alone tool in modern practice.

In children, our results showed that fever, anorexia, and nausea/vomiting were more common and more strongly associated with histologically proven appendicitis. However, the diagnostic accuracy of the Alvarado score remained modest, echoing the conclusions of studies that both reported that while sensitivity in pediatric cohorts is relatively high, specificity is insufficient to avoid unnecessary operations without imaging support, as discussed by Pogorelić et al. (2015) [10] and Bai et al. (2023) [9]. Pediatric-focused studies highlight the value of combining clinical scores with ultrasound, particularly to reduce CT exposure in younger populations [11,12]. Our results reinforce this approach, as CT findings were strongly predictive of appendicitis in our cohort, with non-inflamed CT reports carrying a very low odds of histopathological confirmation (OR = 0.16).

Pediatric literature further supports this caution; all found that although Alvarado and the Pediatric Appendicitis Score correlate well with true appendicitis, neither achieves the accuracy needed for definitive decision-making, especially in equivocal cases [13,16,17,21]. Our study, therefore, adds weight to the growing consensus that age-specific diagnostic pathways are necessary. Children may benefit more from adjunctive imaging or pediatric-specific scores, while in adults, integration with AIR or Adult Appendicitis Score models may help improve specificity [14,20,22].

Our findings also resonate with work in specific populations. For example, Kong et al. [18] noted reduced predictive accuracy of Alvarado in South African patients, while Merhi et al. [19] questioned whether clinical judgment may outperform scoring systems in some contexts. Similarly, recent regional studies confirm that combining Alvarado with imaging, such as ultrasound, can substantially reduce false positives according to Al-Wageeh et al. (2024) [15]. The relatively high false-positive rate in our series underscores this need for a combined approach. Our study is not without limitations. Firstly, the retrospective, single-center nature of the study limits the generalizability of our findings and may result in inconsistency in symptom documentation. Secondly, unequal numbers in age groups reduce statistical power in children's grouping. Thirdly, missing histopathology reports in four cases, and low specificity at equivocal cut-offs may increase the risk of false positives, which is an inherent limitation of the tool that may hinder the accuracy of our results. Lastly, we noted that the Alvarado score had a low sensitivity (~50%) at the traditional surgical threshold, which challenges its clinical utility.

## Conclusions

In this single-center cohort, the Alvarado score demonstrated similar discriminative ability in adults and children (AUC 0.74 vs 0.77) and broadly comparable diagnostic performance at standard thresholds across age groups. However, the high threshold (>7) showed clinically low sensitivity (≈50% overall), limiting its usefulness as a stand-alone ‘rule-in’ criterion. Lower thresholds (≥5) increased sensitivity but markedly reduced specificity. These findings support the use of the Alvarado score for initial risk stratification in both adults and children, with diagnostic decisions guided by integration with imaging and clinical judgment.
